# Clinical Rhabdomyolysis With Acute Kidney Injury Secondary to High-Intensity Rosuvastatin Use: A Case Report

**DOI:** 10.7759/cureus.10932

**Published:** 2020-10-13

**Authors:** Deepak Chitralli, Ronak Raheja, Kishore Br

**Affiliations:** 1 Nephrology, Columbia Asia Referral Hospitals Yeshwanthpur, Bengaluru, IND; 2 Internal Medicine, Columbia Asia Referral Hospital Yeshwanthpur, Bengaluru, IND

**Keywords:** statin-induced necrotizing autoimmune myopathy, myopathy, proximal myopathy, myoglobin, intensity of statin dosing

## Abstract

Statins are the primary class of medication used to lower serum cholesterol concentration for both primary and secondary prevention of cardiovascular disease. Muscle pain is a frequent adverse effect of statins. Severe myonecrosis leading to clinical rhabdomyolysis is rare.

We encountered a 63-year-old male with a medical history of hypertension, type 2 diabetes mellitus, and coronary artery disease with angioplasty in 2008 and 2020. He was started on rosuvastatin 40 mg (0-0-1) along with dual anti-platelets post angioplasty and was discharged home. He traveled back to his hometown and noticed progressive symmetric muscle weakness with decreased urine output. After visiting another hospital he presented to us with severe proximal muscle weakness and acute renal failure. Laboratory investigations were initiated which demonstrated clinically significant derangement in serum creatinine phosphokinase, serum creatinine, urine myoglobin along with deranged liver enzymes. He was subjected to nerve conduction studies for his muscle weakness which was normal and electromyography showed abnormal spontaneous muscle activity in all examined muscles (fibrillations, positive sharp waves, and pseudomyotonic discharges) suggestive of an irritable myopathy. The medication was stopped and he was treated with eight cycles of hemodialysis until his muscle weakness and laboratory parameters improved. He was then discharged with some improvement in muscle weakness. On two week follow-up, the patient showed partial improvement after discontinuation of all lipid-lowering medication.

## Introduction

Statins are the primary class of medication used to lower serum cholesterol concentration for both primary and secondary prevention of cardiovascular disease. Muscle pain is a frequent adverse effect of statins with an incidence of about 15% of users taking the medication [1]. It is important for treating physicians to classify candidates for high-intensity statin therapy versus low-intensity statin therapy before initiating treatment. Analyses conducted by the United States Food and Drug Administration have reported the rate of fatal rhabdomyolysis as 0.15 per 1 million statin prescriptions dispensed [1]. Although muscle toxicity remains a concern, severe myonecrosis leading to clinical rhabdomyolysis is rare, affecting perhaps 0.1% of patients [2].

We use this paper to briefly review and differentiate between the possible spectrum of muscle pathologies ranging from myalgia, myopathy, myositis, myonecrosis, and clinical rhabdomyolysis in accordance with the 2014 National Lipid Association definitions. Our goal is to briefly recap the Indications for statin use as per the American Heart Association /American College of Cardiology guidelines and remind treating physicians about considering the intensity of statin therapy that patients are on. We briefly present a case of rosuvastatin-induced myopathy.

## Case presentation

We encountered the case of a 63-year-old male with a past medical history of type 2 diabetes mellitus for 20 years, hypertension for 10 years, and coronary artery disease with percutaneous transluminal coronary angioplasty in 2008. He presented in January 2020 to a nearby hospital with complaints of chest pain where he was evaluated and on angiography was found to have triple vessel disease and appropriate stents were placed on February 5, 2020. He was then discharged home with changed medication. He was started on rosuvastatin 40 mg in place of his 20 mg atorvastatin and other medication changes as shown in Table 1.

**Table 1:** Comparison of new medication post-procedure versus old medication

Old medication (from 2008 to 2020)	New medication (after procedure 2020)
[Atorvastatin 20 mg + aspirin 75 mg combination pill] (0-1-0)	Aspirin 75 mg (0-1-0), ticagrelor 90 mg (1-0-1), rosuvastatin 40 mg (0-0-1)
[Cilnidipine 10 mg + telmisartan 40 mg combination pill] (1-0-0)	[Telmisartan 40 mg + metoprolol 50 combination pill] (1-0-0)
[Metoprolol succinate 50 mg] (0-0-1)	Cilnidipine 10 mg twice daily (1-0-1)
Glyceryl trinitrate 2.5 mg as needed for chest pain	Stopped
Titrated insulin and metformin variably	Tablet gliclazide 5 mg (1-0-1), tablet vildagliptin 50 mg 1-0-1 mixture Insulin (30/70) 16-0-16 units	

Post-procedure after a few medication changes, he traveled back to his hometown and first noticed progressive symmetric muscle weakness. At first, he was not able to walk or rise from his bed. He did not report any sensory loss or difficulty swallowing. He reported worsening fatigue, weakness, nausea, and decreasing urine output. He presented to his primary hospital where he was found to have acute kidney injury with a serum creatinine of 6.4 with hyperkalemia.

He then presented to our hospital where physical examination showed preserved higher mental functions, intact cranial nerves with visual defects, and normal sensory findings with reduced proximal muscle strength symmetrically in both upper and lower limbs (2/5) without any weakness of distal muscles in both upper and lower limbs strength (5/5). There were no fasciculations, swelling of the affected muscles, or skin rash. Muscle bulk was unaffected (4/5). Reflexes were universally weak. Other systems were within normal limits.

**Figure 1:**
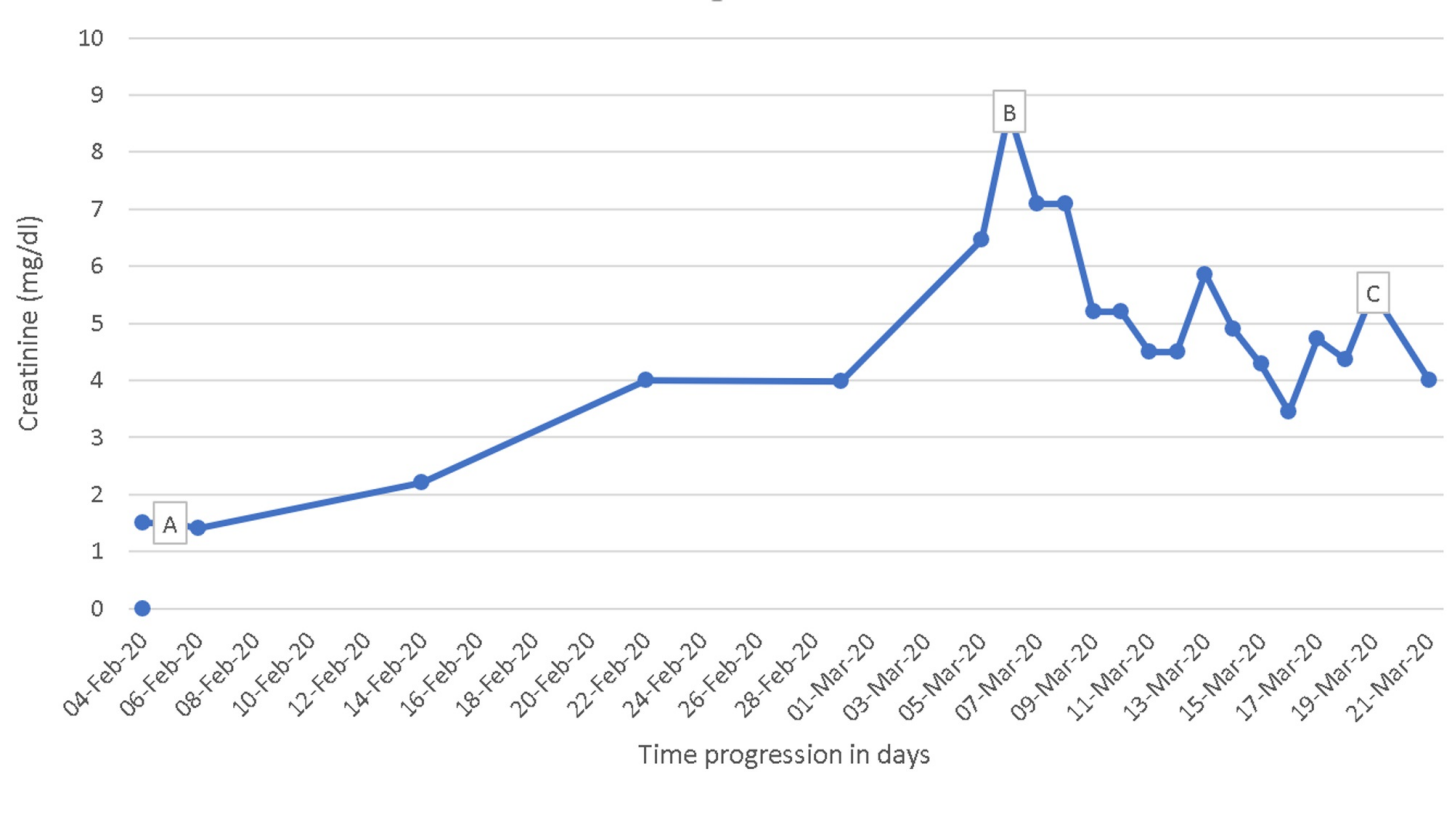
Creatinine level variation (mg/dl) (A) The date of coronary stent placement: 06/Feb/2020 (creatinine: 1.50); (B) the date he presented to our hospital: 07/Mar/2020 (creatinine: 8.71); (C) the date he was discharged home: 21/Mar/2020 (creatinine: 5.54).

The course of progression of muscle weakness in relation to his serum creatinine phosphokinase levels and serum creatinine levels has been tabulated in Table 2. In view of severe renal failure and oliguria, he was initiated on hemodialysis through a temporary hemodialysis catheter and he was dialyzed on a schedule as shown in Table 1. Even after stopping rosuvastatin, he continued to be dialysis dependant for the next two weeks. His creatine phosphokinase levels steadily came down and his urine output gradually started improving after two weeks. His myopathy also improved significantly and he was discharged. On follow-up, his creatinine levels came to 3.8 mg/dl (Figure 1) and he was weaned of hemodialysis. He was also advised not to take statins and discuss with his cardiologist for shifting him to an alternative lipid-lowering medication in the future if the need should arise.

**Table 2:** Progression in regard to creatinine (mg/dl) and CPK levels CPK: creatinine phosphokinase levels. Muscle strength was graded in accordance with the medical research council (MRC grading) as following: 1 - slight flicker movements scene in the muscle group; 2 - muscle contraction present but with gravity eliminated; 3 - muscle contraction present and contracts against gravity; 4 - muscle contraction present against some resistance; 5 - normal and strong muscle contraction.

Date	Event	Creatinine (mg/dl) reference (0.5-1.2 mg/dL)	CPK (IU) reference (40-400)	Power (out of 5)
04/Feb/2020	One day before the procedure (the last dose of atorvastatin 20 mg was given)	1.5		Power
05/Feb/2020	Day of the procedure	1.5		Power 5/5
06/Feb/2020	One day after the procedure (rosuvastatin 40 mg was initiated)	1.4		Normal power 5/5
14/Feb/2020	One-week post-procedure	2.2		Proximal muscle weakness first experienced power 4/5
22/Feb/2020	Two weeks post-procedure	4.00		Weakness progressing power 3/5
29/Feb/2020	Three weeks post-procedure	3.98		Persistent weakness power 2/5
05/Mar/2020	Presented to our hospital	6.46		Persistent weakness power 2/5
06/Mar/2020	Day 1 at our center rosuvastatin 40 mg was discontinued	8.71	1,30,365	Persistent weakness power 2/5
07/Mar/2020	Day 2/cycle 1 of hemodialysis	7.09		Persistent weakness power 2/5
08/Mar/2020	Day 3/cycle 2 of hemodialysis			Persistent weakness power 2/5
09/Mar/2020	Day 4	5.21	60,111	Persistent weakness power 2/5
10/Mar/2020	Day 5 cycle 3 of hemodialysis			Persistent weakness power 2/5
11/ Mar/2020	Day 6	4.50	29,707	Persistent weakness power 2/5
12/Mar/2020	Day 7/cycle 4 of hemodialysis	5.86		Persistent weakness power 2/5
13/Mar/2020	Day 8 at our hospital	4.90		Persistent weakness power 2/5
14/Mar/2020	Day 9/cycle 5 of hemodialysis	4.28	28,502	Gradual improvement power 3/5
15/Mar/2020	Day 10/cycle 6 of hemodialysis	3.44		Gradual improvement power 3/5
16/Mar/2020	Day 11 at our hospital	4.73		Gradual improvement power 3/5
17/Mar/2020	Day 12/cycle 7 of hemodialysis	4.36	5321	Gradual improvement power 3/5
18/Mar/2020	Day 13/cycle 8 of hemodialysis	5.54		Gradual improvement power 3/5
19/Mar/2020	Day 14/discharged home with advice to follow-up regular hemodialysis			Gradual improvement power 3/5
21/Apr/2020	Lab test near home after intermittent hemodialysis	4.00		Weakness improved significantly (4/5)
27/Apr/2020	Phone follow-up	2.80		Weakness Improved significantly (4/5)
04/july/2020	Phone follow-up			Weakness improved to (5//5)

## Discussion

Muscle syndromes associated with statins vary from myalgias, myopathy, myositis, and muscle injury. Drug-induced myopathy is among the most common causes of muscle disease. It ranges from mild myalgias with or without weakness to chronic myopathy with severe weakness and can also cause massive rhabdomyolysis with acute renal failure. Below we have compiled a table (Table 3) to help review muscle pathologies as defined by the 2014 National Lipid Association Statin Muscle Safety Task Force.

**Table 3:** Muscle pathologies overview CK: creatine kinase.

Muscle pathologies overview	
Myalgia	Muscle-discomfort (symptoms): aches, soreness, stiffness, tenderness, or cramps associated with movement. Normal CK level. Myalgia symptoms can be described as similar to what would be experienced with a viral syndrome such as influenza.
Myopathy	Muscle weakness (not due to pain), with or without an elevation in CK level.
Myositis	Muscle inflammation.
Myonecrosis	Elevation in muscle enzymes compared with either baseline CK levels or the upper limit of normal that has been adjusted for age, race, and sex.
Mild myonecrosis	3- to 10-fold elevation in CK.
Moderate myonecrosis	10- to 50-fold elevation in CK.
Severe myonecrosis	50-fold or greater elevation in CK.
Clinical rhabdomyolysis	Myonecrosis + myoglobinuria or myonecrosis + acute renal failure (an increase in serum creatinine of at least 0.5 mg/dL).

Overall, the Statin Safety Expert Panel reaffirms the general safety of statin therapy. It is the belief of the Panel members that in most patients requiring statin therapy that the potential benefits of statin therapy outweigh the potential risks [3].

Mechanism of statin-induced myopathy

Statin-associated myopathy demonstrates that a single agent may cause myopathy by more than one mechanism: (i) autoimmune mechanisms; (ii) direct myotoxicity - on histopathological examination vacuolated muscle fibers may occur with the use of statins; (iii) genetic factors - a trial of statins found an association between a specific solute carrier organic anion transporter 1B1 (SLCO1B1) variant (SLCO1B1*5) and an increased risk of mild adverse events [4].

In a study among 2,52,460 patients treated with lipid-lowering agents, 24 cases of hospitalized rhabdomyolysis occurred during treatment [5]. The American Heart Association /American College of Cardiology 2013 makes a person eligible for statin therapy by recommending it for those as listed in Table 4 [6-8].

**Table 4:** Indications for lipid-lowering medication as per AHA AHA: Ameican heart association, ASCVD: atherosclerotic cardiovascular disease, LDL C: low-density lipoprotein C.

Indications for statin use
ASCVD
LDL C > 190 mg/dL
Primary prevention in type 2 diabetics (age between 40 and 75 years) with LDL C 70 to 189 mg/dL
Primary prevention of estimated 10-year risk of ASCVD > 7.5% and age 40 to 75 years

Very elderly people pose a troubling dilemma for the cardiovascular community, guideline writers, and clinical practitioners. Although they are at high risk of a cardiovascular event by virtue of their age alone, evidence of statin use is very limited in this age group [9]. The statin-associated muscle symptoms scoring system clinical index (SAMS-CI), was proposed by the expert task force for evaluating statin-associated symptoms [9]. We used the SAMS-CI which demonstrated a high probability for statin-induced muscle symptoms.

Although there are many guidelines for the management of statin therapy, the concept of the maximum tolerated statin therapy is of extreme importance and has been added to the American College of Cardiology 2018 guidelines. We recommend treating physicians consider the intensity of dosing statin medication carefully. We have summarized the aspects of dosing statin medication in Table 5. It is also noteworthy to discuss that the concept of treating to target low-density lipoprotein levels (LDL) has been removed by the task force.

**Table 5:** Dosing of statin summary AHA: Ameican Heart Association, ASCVD: atherosclerotic cardiovascular disease, LDL C: low-density lipoprotein C.

Dosing type	High intensity	Moderate intensity	Low intensity
Aim	On average lowers LDL C by more than 50%	On average lowers LDL C between 30% and 50%	On average lowers LDL C less than 30%
Choice of drugs	Include atorvastatin (40-80 mg) rosuvastatin (20-40 mg)	Include atorvastatin (10-20mg) rosuvastatin (5-10 mg)	--
Choice of drugs	--	Simvastatin (20-40), pravastatin (40-80), lovastatin (40 mg), fluvastatin (80 mg), pitavastatin (2-4 mg)	Simvastatin (up to 10 mg), pravastatin (up to 20 mg), lovastatin (up to 20 mg), fluvastatin (up to 40 mg), pitavastatin (up to 1 mg)
Indications			
If LDL C > 190	First choice if age below 75 years	If unable to tolerate high dose or age >75 years	If unable to tolerate a moderate dose
If atherosclerosis ACS/stroke/vascular procedures	First choice if age below 75 years	If older than 75 years or unable to tolerate a high dose	Only if unable to tolerate a moderate dose
Diabetics	First choice in diabetics with ASCVD risk >7.5%	First choice in diabetics with ASCVD risk less than 7.5%	If unable to tolerate the moderate dose
Nondiabetics	First choice if ASCVD risk >7.5%	Optional consider if ASCVD risk <7.5%	If unable to tolerate

**Figure 2:**
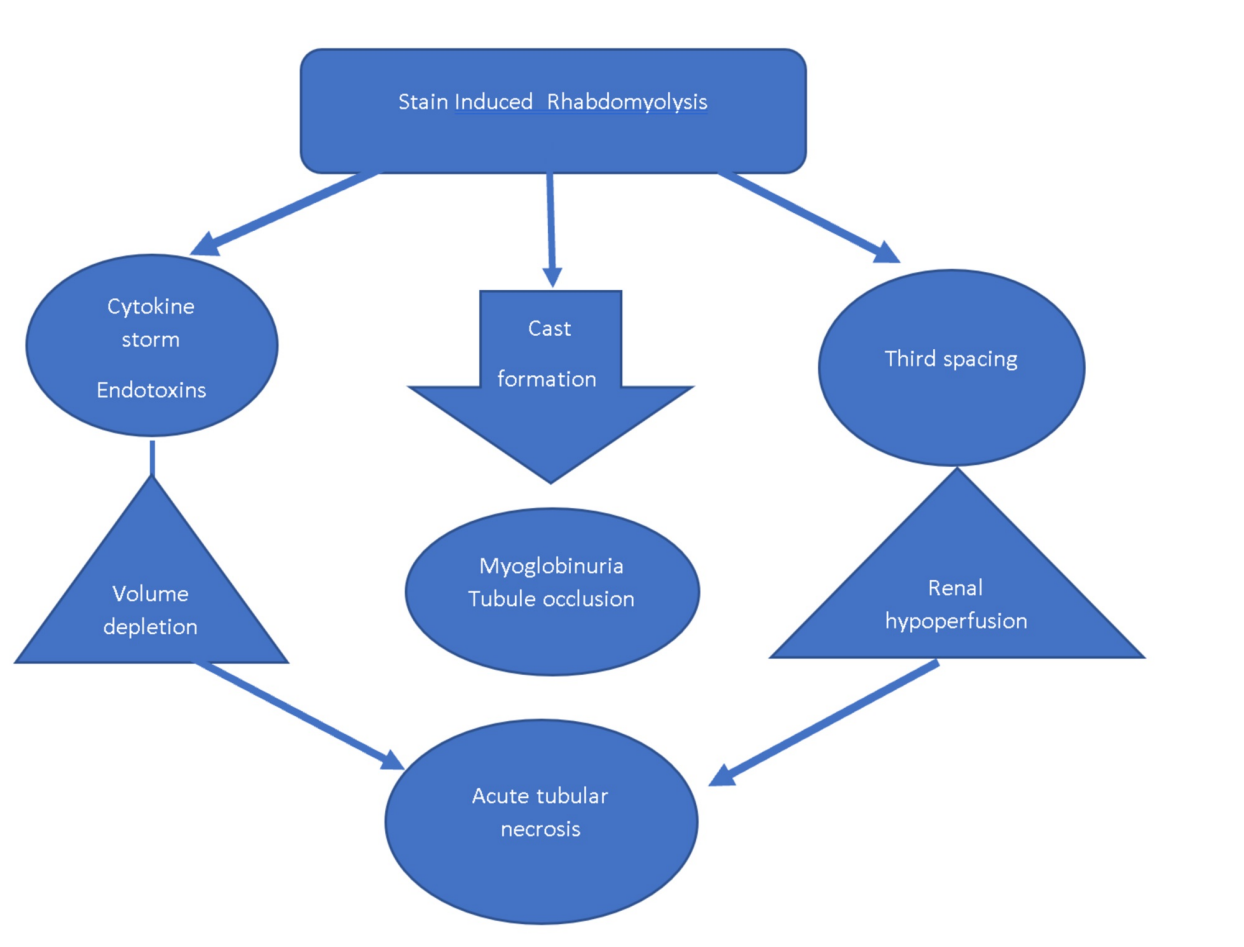
Mechanism of statin-induced acute kidney injury

It is important to rule out the obvious causes of rhabdomyolysis such as trauma, hyperthermia, severe metabolic derangements, high-dose steroid use, and congenital myopathies including mitochondrial diseases. Glucocorticoid-induced myopathy is usually painless. Most other myopathies have some element of pain and tenderness involved. In polymyalgia rheumatica, the erythrocyte sedimentation rate is increased but creatinine kinase levels are usually not significantly elevated. In inflammatory myopathies, there will be elevation in both erythrocyte sedimentation rate and creatinine phosphokinase levels. The mechanism of statin-induced rhabdomyolysis has been schematically illustrated in Figure 2.

In statin-induced myopathy usually, the erythrocyte sedimentation rate will be normal, but creatinine phosphokinase will be elevated. In the approach to weakness, we should use a systematic flow to identify the site of lesion as either upper motor neuron, lower mortar neuron, nerve end terminals, neuromuscular junction, or the muscle itself. Then, we should consider electrolyte imbalances or poisons. 

Management of statin-induced muscle pathology should be guided by the extent of creatinine phosphokinase elevation. If creatinine phosphokinase is elevated to more than 10 times, then the upper normal limit indicates stopping the medication, stabilizing the patient even to the extent of hemodialysis to manage acute kidney injury. After discontinuation of statin therapy, we recommend repeating the lipid profile on a later date and switching to a low-dose alternative lipid-lowering therapy regimen.

## Conclusions

It is important to identify statin-induced myonecrosis. It is also important for the general physician to consider dosing statin carefully. It may be advisable to educate patients on statin-induced side effects when using a high dose. We attempted to review the spectrum of myopathies and indications for statin use. Stains may rarely show clinical rhabdomyolysis and it should always be considered as a differential diagnosis in patients presenting with weakness and renal failure following a cardiac intervention. Careful analysis of drug history will help in early diagnosis and prompt treatment of statin-induced myopathy. Stain-induced myopathy usually indicates stopping the medication and possibly switching to an alternative medication or regimen. It should take approximately four months for rosuvastatin-induced rhabdomyolysis to improve if appropriate interventions are taken. 
